# Whitening Activity of Constituents Isolated from the *Trichosanthes* Pulp

**DOI:** 10.1155/2020/2582579

**Published:** 2020-07-22

**Authors:** Rongchao Zhang, Xiuqin Hu, Bo Zhang, Zhen Wang, Chunxiang Hao, Jie Xin, Qingmei Guo

**Affiliations:** ^1^Lunan Engineering Technology Research Center for the Development of Traditional Chinese Medicine Resources of ShandongProvince, School of Pharmacy, Linyi University, Linyi 276000, China; ^2^School of Pharmacy, Shandong University of Traditional Chinese Medicine, Jinan 250355, China

## Abstract

Whitening cosmetics market has a bright future, and pure natural whitening products of traditional Chinese medicine have always been a research hotspot. In this research, the whitening active ingredient of Chinese medicine *Trichosanthes* pulp was isolated and purified for the first time, and its whitening mechanism was clarified. Chromatographic methods such as silica gel, ODS, and HPLC were used to isolate and purify them. B16 cells were used to measure the antioxidant activity, tyrosinase activity, and melanin removal activity. A total of 20 compounds were isolated, including *p*-hydroxybenzaldehyde (1), salicylic acid (2), vanillic acid (3), isovanillic acid (4), protocatechuate (5), *trans*-cinnamic acid (6), 4-coumaric acid (7), *trans*-ferulic acid (8), drechslerol-B (9), cyclotucanol 3-palmitate (10), 5-acetoxymethyl-2-furaldehyde (11), 5-hydroxymethylfurfural (12), diosmetin (13), apigenin (14), chrysoeriol (15), luteolin (16), 4′-hydroxyscutellarin (17), quercetin (18), 3′,5-dihydroxy-7-(*β*-D-glucopyranosyloxy)-4′-methoxyflavone (19), and cofloxacin-7-O-*β*-D-glucoside (20). Among them, compounds 9, 10, 11, 12, 13, 14, 15, 16, 17, 18, 19, and 20 have good antioxidant repairing effects; compounds 3, 4, 5, 6, and 7 have high black inhibition; compounds 1, 2, 3, 4, 5, 6, 7, 8, 11, 12, 13, 14, 15, 16, 17, 18, 19, and 20 have obvious tyrosine acidase inhibitory activity. The results laid foundation for the further development and utilization of *Trichosanthes* pulp resources and also provide a basis for the development of natural whitening cosmetics.

## 1. Introduction

*Trichosanthes kirilowii* Maxim. is a perennial climbing herb of the Cucurbitaceae family. Its fruit, seeds, peel, and root are all commonly used as traditional Chinese medicines. For a long time, the pulp of *Trichosanthes* is often discarded in the production and processing of Semen Trichosanthis and Pericarpium Trichosanthis, resulting in significant waste. At present, reports on *Trichosanthes* pulp are mainly focused on the content comparison of the sugar, amino acids, vitamins, and other nutrients [1–4]. There is no report on the chemical composition of *Trichosanthes* pulp. Therefore, it is necessary to study the chemical constituents of the pulp systematically.

At the same time, with the rapid development of the economy, cosmetics are not only high-consumption lifestyle products but also become the most popular “necessities” for women. Many classical Chinese medicine books or materia medica has recorded that *Trichosanthes* pulp has the function of whitening and wrinkle removing effects [5–7]. The previous results of our research group show that the water extract of *Trichosanthes* pulp had higher tyrosinase inhibitory activity, and the tyrosinase inhibitory rate was about 70%. The common whitening agents vitamin C (VC) and arbutin were used as control substances [8]. It was preliminarily proved that the extract of *Trichosanthes* pulp had a certain whitening effects by inhibiting the activity of tyrosinase. Therefore, it is of great significance to study the chemical constituents and whitening mechanism of *Trichosanthes* pulp.

The whitening mechanism can be summarized as inhibiting the synthesis of melanin and accelerating the transfer of melanin. The process of melanin formation in melanocytes is as follows: tyrosinase oxidizes tyrosine to polymorph BA (DOPA); then it is oxidized to dopaquinone. After a series of metabolic reactions, melanin is produced. Tyrosinase is the only rate-limiting enzyme in the reaction process, and its activity determines the amount of melanin synthesized. When tyrosinase activity is enhanced, melanin synthesis increased; when tyrosinase activity is inhibited, melanin synthesis is reduced [9]. The active center of tyrosinase has a double copper active site, and each copper ion combines with 3 histidine residues with 1 or 2 valences respectively. Therefore, the reduction with antioxidant effects can interact with the copper atom on the active site of tyrosinase or reduce the intermediate in the process of melanin synthesis to inhibit the formation of melanin [10, 11]. In addition, inhibiting the transfer of mature melanocytes to keratinocytes can also play a role in whitening.

Cell culture technology is used to determine the effect of whitening active ingredients on tyrosinase activity and melanin production in melanocytes in vitro. The mouse melanoma cell line (B16 cells) is derived from mouse skin melanoma cells, and its basic structure, especially regarding melanin synthesis, is basically the same as that of human normal melanoma cells. Since it is very difficult to culture human primary skin melanoma cells, the mouse model can be used as a test cell for the determination of whitening active ingredients. Therefore, B16 cells were used to measure the antioxidant activity, tyrosinase activity, and melanin removal activity, and the whitening active components and the related mechanism were determined preliminarily. The results laid foundation for the further development and utilization of *Trichosanthes* pulp resources and also provide a basis for the development of natural whitening cosmetics.

## 2. Experiments

### 2.1. Materials

*Trichosanthes trichosanthis* was collected from the Hebao herbal medicine planting base in Pingyin, Jinan. The samples were identified by Professor Qingmei Guo of Shandong University of Traditional Chinese Medicine. After removing the peel and seeds, the pulp part of the fruit was obtained. After freeze-drying, the pulp was crushed, mixed, and finally placed in a desiccator for further use.

### 2.2. Cell Viability

B-16 cells (mouse melanoma cells) were purchased from KeyGEN and maintained in DMEM medium supplemented with 10% fetal bovine serum (FBS), at 37°C in a humidified atmosphere with 5% CO_2_. Then, B-16 cells were seeded in 96-well plates (10 × 104 cells/well) for 24 h incubation.

### 2.3. Preparation of Different Extracts

The raw material of the pulp of *Trichosanthes kirilowii* was 4.5 kg. The pulp was soaked in 10 times the amount of 95% ethanol 3 times. The extracts were combined and concentrated to about 3.0 L. An ethyl acetate extract was obtained by dispersing the ethanol extract into 15 L water, extracting it with ethyl acetate until the extract was colorless. The extracts were combined and concentrated under reduced pressure.

The ethyl acetate extract was mixed with silica gel (100–200 mesh) at a ratio of 1 : 2 (extract: silica gel, v/v). The solvent was evaporated, and the gel was reserved. The glass chromatography column (80 mm × 1200 mm) was packed with 200–300 mesh silica gel and D101 macroporous resin, and then the column was eluted with ethyl acetate, petroleum ether (10%, 25%, 50%, 75%, and 100%), and methanol, ethyl acetate (5%, 10%, 25%, 50%, 75%, and 100%), to collect each flow. Finally, each fraction was collected and configured to the following concentrations: 0.003125 mg/mL, 0.00625 mg/mL, 0.0125 mg/mL, 0.025 mg/mL, 0.05 mg/mL, 0.1 mg/mL, 0.25 mg/mL, 0.5 mg/mL, 1.0 mg/mL, and 2.0 mg/mL, and then used to process the cultured cells. At the end of treatment, 20 *μ*L of MTT solution (5 mg/mL) was added into each well. The cells were incubated 20 min at 37°C. The supernatant was discarded, and 100 *μ*L of DMSO was added to each well. The absorbance was measured at 490 nm. The experiment was repeated 3 times. The survival rate was calculated as follows: survival rate% = (experimental group-blank group)/(control group -blank group) × 100%. The optimal concentration (C1) of each fraction was calculated according to the functional relationship between the survival rate and each fraction concentration. C1 was diluted 2, 4, 6, and 8 times to obtain the concentrations C2, C3, C4, and C5, respectively. The different extracts of the pulp are shown in Table 1.

### 2.4. Whitening Experiment

#### 2.4.1. Antioxidant Test

An MTT assay was used to determine cell viability (MTT Kit, Kaiji Biology). Then, B-16 cells were seeded in 96-well plates (10 × 10^4^ cells/well) for 24 h and treated with different extracts or compound for 24 h. After treatment, the culture medium was discarded and washed twice with PBS. Then, 0.05% H_2_O_2_ was added, and the cells were cultured for 4 h. After that, B-16 cells were incubated in 20 *µ*L MTT solution (5 mg/mL) at 37°C for 4 h. Then, the culture supernatant was removed, and the residue was dissolved in 100 *µ*L DMSO. The absorbance was detected at 490 nm using a microplate reader (Tecan Trading AG). The experiments were performed in parallel 3 times.

#### 2.4.2. Inhibition of Melanin Production

The production of melanin was determined by detecting melanin content in cells by NaOH pyrolysis [12]. Cells were seeded in 96-well culture plates for 24 h at a cell density of 10 × 104 cells/well. Then, the cells were treated with different extracts or compound for 24 h. After that, the cell culture supernatant (300 *µ*L) was removed, and the residue was dissolved in NaOH (1 mL, 1.0 mol/L) for 24 h at room temperature. The absorbance was measured at 475 nm, using a microplate reader (Tecan Trading AG). The experiments were performed in parallel with 3 replicates. The IC_50_ was determined by GraphPad Prism 5 software (GraphPad Software, Inc., San Diego, California), and arbutin was used as a positive control (IC_50_ = 15.6 *µ*L).

#### 2.4.3. Tyrosinase Activity Determination

The cell culture supernatant was removed, and the residue was dissolved in Tritonx-100 (90 *µ*L) and tyrosinase (10 *µ*L, 1.0 mg/mL, Sigma, T3824 25ku). After blending, the mixture was incubated at 37°C for 30 min. The absorbance was measured at 475 nm using a microplate reader (Tecan Trading AG). The experiments were performed in parallel 3 times. The IC50 was determined by GraphPad Prism 5 software (GraphPad Software, Inc., San Diego, California), and arbutin was used as a positive control (IC50 = 15.6 *µ*L).

### 2.5. Extraction and Isolation

The ethyl acetate extract was dissolved in methanol, and the column was packed with 200–300 mesh silica gel, which was equilibrated by petroleum ether. Then, a gradient elution consisting of petroleum ether and ethyl acetate in ratios of 2 : 1, 1 : 1, and 1 : 2; ethyl acetate; ethyl acetate and methanol in ratios of 2 : 1, 1 : 1, and 1 : 2; and methanol was used in conjunction with TLC to obtain compounds A (11.5 g), B (10.4 g), C (12.1 g), D (5.2 g), and E (6.1 g).

A was separated by polyamide column chromatography, using dichloromethane and methanol in ratios of 2 : 1, 1 : 1, and 1 : 2 as the elution solvent. The eluate was monitored by TLC. Spots with the same retention factor (rf) were combined, and A1 and A2 were obtained. Then, A1 was prepared by TLC, using dichloromethane and methanol in a ratio of 1 : 1 as the developing agent, and purified several times by the gel column. Finally, compound 1 (17 mg) and compound 2 (27 mg) were obtained. A2 was purified by silica gel column chromatography, using dichloromethane and methanol as the eluent in ratios of 1 : 1 and 1 : 2. Compound 3 (11 mg), compound 4 (12 mg), and compound 5 (23 mg) were obtained. B was separated by reversed-phase C18 medium pressure chromatography (RP-C18), using methanol, water in a gradient elution as follows: 0%,10%, 15%, 20%, 25%, 30%, 35%, 40%, 50%, 75%, 90%, and 100%. The flow rate was 10 mL·min^−1^. The flow fractions with the same rf were combined, and B1 and B2 were obtained. B1 was repeatedly purified by the gel column, with methanol as an elution solvent. Compounds 6 (24 mg), 7 (21 mg), and 8 (18 mg) were obtained after repeated elution. B2 was purified by the gel column and methanol, and compound 9 (11 mg) was obtained. C was separated by RP-C18 and eluted by methanol and water with a flow rate of 10 mL·min^−1^. The flow fractions with the same rf were combined, and C1, C2, and C3 were obtained. C1 was prepared by the gel column and eluted by methanol, and compound 10 (13 mg) was obtained. C2 was separated by silica gel column and eluted by ethyl acetate and methanol, with a gradient elution in ratios of 2 : 1, 1 : 1, and 1 : 2. C2 was purified by preparative liquid phase extraction, and compounds 11 (15 mg) and 12 (14 mg) were obtained. C3 was separated by the polyamide column, eluted by methanol and water, and purified by HPLC. Compounds 13 (34 mg), 14 (42 mg), and 15 (38 mg) were obtained. D was separated by RP-C18 and eluted with methanol and water. The flow rate was 10 mL·min^−1^. Compounds 16 (36 mg), 17 (19 mg), and 18 (37 mg) were obtained by HPLC. Compounds 19 (29 mg) and 20 (34 mg) were obtained by gradient elution using ethyl acetate and methanol in ratios of 1 : 1 and 1 : 2, followed by 100% methanol on a silica gel column.

## 3. Results and Discussion

### 3.1. Determination of Active Extracts (E1–E11)

**Figure 1:**
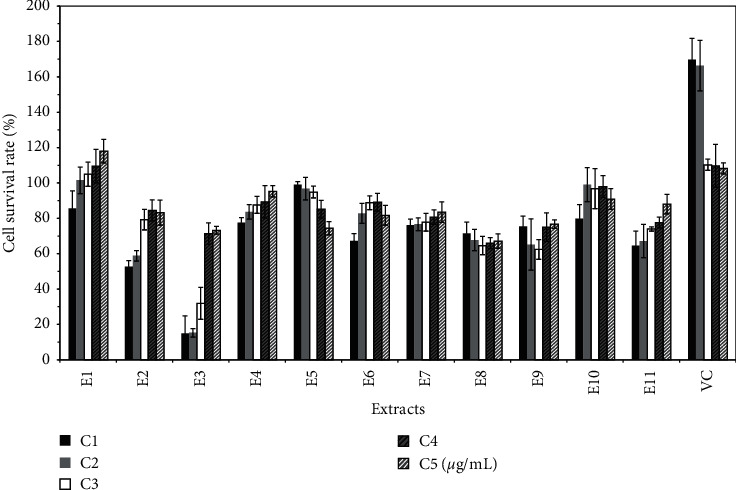
Cell survival rate (%) of the antioxidant test of polar extracts.

**Figure 2:**
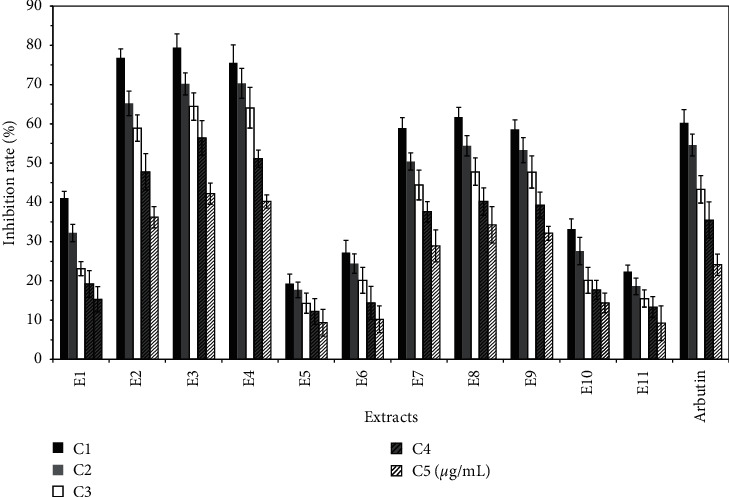
Effect of different extracts on the inhibition rate of melanin synthesis in B16 cells ($\bar{X}\pm S$%).

**Figure 3:**
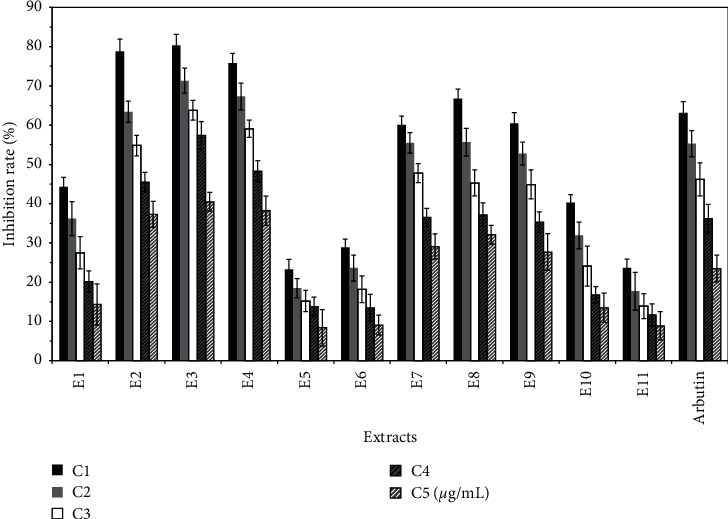
Effect of different extracts on the inhibition rate of tyrosinase in B16 cells ($\bar{X}\pm S$%).

The optimal concentration (namely, C1) of each extract was calculated and is shown in Table 1. The antioxidant experiment shows that all extracts have antioxidant repair effect, but compared with VC, the antioxidant effect of each group is weak. The antioxidant activity of E1, E3, E4, E7, and E11 decreased with increasing concentration, as shown in Figure 1. The melanin inhibition test showed that all polar components in the pulp of *Trichosanthes kirilowii* inhibited melanin synthesis. In particular, the melanin inhibition rate was significantly higher for E2, E3, and E4 than that of the positive control arbutin, as shown in Figure 2. The results of tyrosinase activity show that all polar components in the pulp of *Trichosanthes kirilowii* inhibited tyrosinase activity, and the inhibition trend was similar to that of melanin inhibition. The inhibition of tyrosinase activity was significantly higher for E2, E3, E4, and E8 than that of the positive control. In general, the extracts from petroleum ether have a low inhibitory effect on tyrosinase. The extracts from ethyl acetate have a high inhibitory effect on tyrosinase, especially the polar components from ethyl acetate. The extracts from n-butanol also have a high inhibitory effect on tyrosinase, as shown in Figure 3.

### 3.2. Isolation and Identification of Monomer Components

20 compounds were separated by silica gel and ODS chromatogram columns as well as preparative HPLC. On the basis of NMR and MS data analysis, their structures were elucidated. The 20 compounds are shown in Table 2, and the specific analytical data of monomers are shown in Appendix 1.

### 3.3. Validation Analysis of Effective Compounds

#### 3.3.1. Validation Analysis of Antioxidant Compounds

E1, E2, E3, E4, and E5 have antioxidant effects. The main compounds isolated from the above components were drechslerol-B (compound 9), cyclotucanol 3-palmitate (compound 10), 5-acetoxymethyl-2-furaldehyde (compound 11), 5-hydroxymethylfurfural (compound 12), diosmetin (compound 13), apigenin (compound 14), chrysoeriol (compound 15), luteolin (compound 16), 4′-hydroxyscutellarin (compound 17), quercetin (compound 18), 3′,5-dihydroxy-7-(*β*-D-glucopyranosyloxy)-4′-methoxyflavone (compound 19), and cofloxacin-7-O-*β*-D-glucoside (compound 20).

**Figure 4:**
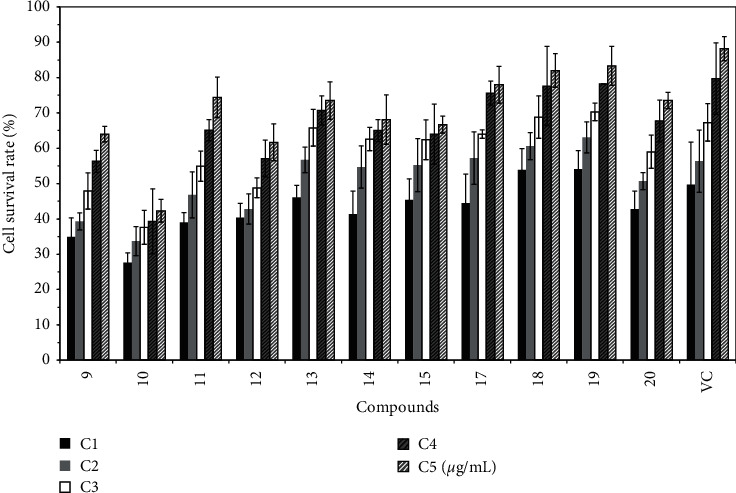
Cell survival rate (%) of the antioxidant experiment of different compounds from *Trichosanthes* extracts.

The antioxidant repair activity of the above compounds was determined according to the method in Section 2.4.1. VC was used as the control, and the concentrations of compounds were 100 *µ*g/mL, 200 *µ*g/mL, 400 *µ*g/mL, 800 *µ*g/mL, and 1000 *µ*g/mL. Each concentration was analyzed 3 times. The survival rate was calculated. The results show that components 1, 2, 3, 4, and 5 had good antioxidant activities. The main compounds were glycosides, indicating that glycosides have good antioxidant activities. Phenolic acids, such as 5-acetoxymethyl-2-furaldehyde (compound 11), 5-hydroxymethylfurfural (compound 12), diosmetin (compound 13), apigenin (compound 14), chrysoeriol (compound 15), luteolin (compound 16), 4′-hydroxyscutellarin (compound 17), quercetin (compound 18), and 3′,5-dihydroxy-7-(*β*-D-glucopyranosyloxy)-4′-methoxyflavone, also have good antioxidant activities, due to the phenolic hydroxyl group and unsaturated double bonds [26, 27]. The results are shown in Figure 4.

#### 3.3.2. Validation Analysis of Melanin Inhibition Compounds

E11, E12, E13, E16, E17, and E18 have good inhibitory effects on melanin synthesis. The main compounds isolated from the above components are shown in Table 2. According to the method in Section 2.4.2, the effect of each compound on the synthesis of melanin was determined. Each compound was evaluated at the following concentrations: 1000 *µ*g/mL, 800 *µ*g/mL, 400 *µ*g/mL, 200 *µ*g/mL, and 100 *µ*g/mL. Each measurement was performed in triplicate, and the inhibition rate of melanin synthesis was calculated.

**Figure 5:**
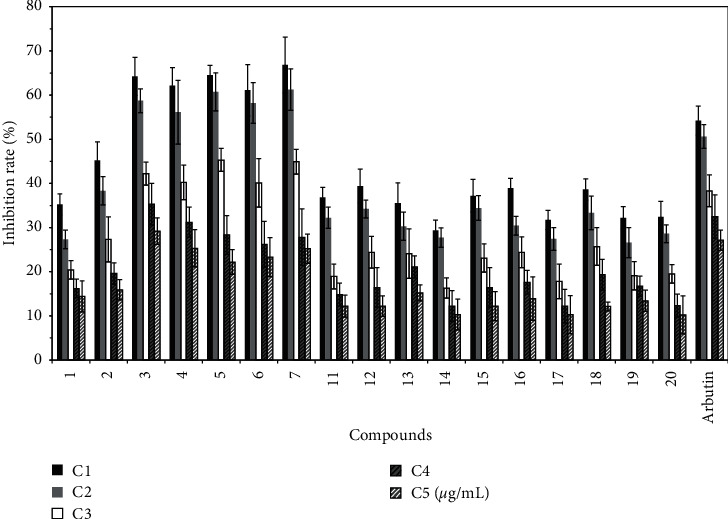
Inhibition rate of different compounds on melanin synthesis in B16 cells ($\bar{X}\pm S$%).

Protocatechuate (compound 5), 5-acetoxymethyl-2-furaldehyde (compound 11), 5-hydroxymethylfurfural (compound 12), diosmetin (compound 13), p-hydroxybenzaldehyde (compound 1), salicylic acid (compound 2), vanillic acid (compound 3), isovanillic acid (compound 4), *trans*-cinnamic acid (compound 6), 4-coumaric acid (compound 7), apigenin (compound 14), chrysoeriol (compound 15), luteolin (compound 16), 4′-hydroxyscutellarin (compound 17), quercetin (compound 18), 3′,5-dihydroxy-7-(*β*-D-glucopyranosyloxy)-4′-methoxyflavone (compound 19), cofloxacin-7-O-*β*-D-glucoside (compound 20) can inhibit melanin production [28]. In particular, vanillic acid (compound 3), isovanillic acid (compound 4), protocatechuate (compound 5), *trans*-cinnamic acid (compound 6), and 4-coumaric acid (compound 7) have high melanin inhibition. The results are shown in Figure 5.

#### 3.3.3. Verification and Analysis of Compounds Inhibiting Tyrosinase Activity

**Figure 6:**
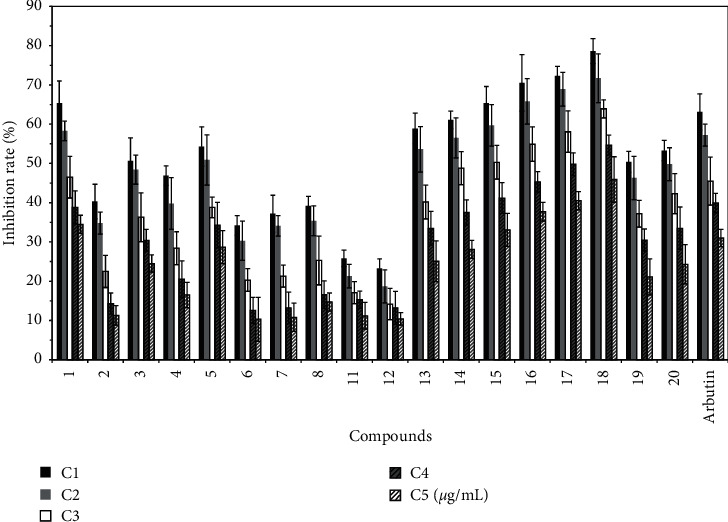
Inhibition rate of different compounds on tyrosinase activity of B16 cells ($\bar{X}\pm S$%).

E2, E3, E4, E7, E8, and E9 have good inhibitory effects on tyrosinase activity. The main compounds isolated from the above components are shown in Table 2. According to the method presented in Section 2.4.3, arbutin was used as the control, and the compound concentration was 1000 *µ*g/mL, 800 *µ*g/mL, 400 *µ*g/mL, 200 *µ*g/mL, and 100 *µ*g/mL. Each measurement was performed 5 times, and the inhibition rate of tyrosinase was calculated. The results are shown in Figure 6.

The results show that diosmetin (compound 13), apigenin (compound 14), chrysoeriol (compound 15), luteolin (compound 16), 4′-hydroxyscutellarin (compound 17), quercetin (compound 18), 3′,5-dihydroxy-7-(*β*-D-glucopyranosyloxy)-4′-methoxyflavone (compound 19), cofloxacin-7-O-*β*-D-glucoside (compound 20), p-hydroxybenzaldehyde (compound 1), salicylic acid (compound 2), vanillic acid (compound 3), isovanillic acid (compound 4), protocatechuate (compound 5), *trans*-cinnamic acid (compound 6), 4-coumaric acid (compound 7), and *trans*-ferulic acid (compound 8) all have obvious tyrosinase inhibitory activities.

Hydroxyl substituted compounds on the benzene ring, such as p-hydroxybenzaldehyde (compound 1), salicylic acid (compound 2), vanillic acid (compound 3), isovanillic acid (compound 4), protocatechuate (compound 5), *trans*-cinnamic acid (compound 6), 4-coumaric acid (compound 7), *trans*-ferulic acid (compound 8), and flavonoids, exhibited high tyrosinase inhibition, and the inhibition rate of parasubstituted compounds was significantly higher than that of orthosubstituted compounds. For example, the inhibitory activity of p-hydroxybenzaldehyde was significantly higher than that of salicylic acid, and the inhibitory activity of vanillic acid was significantly higher than that of isovanillic acid. Moreover, the inhibitory activity of p-hydroxybenzaldehyde was significantly higher than that of salicylic acid, vanillic acid, isovanillic acid, and protocatechuate. The reason may be that the nucleophilic groups around the active center of tyrosinase, such as –SH, −NH2, and –OH, occupy the space around the active center and thus prevent tyrosine from attacking the active center. Finally, the synthesis of melanin was inhibited [28]. The inhibitory activity of compounds containing o-dihydroxy was stronger than that of parahydroxy compounds, and the inhibitory activity of protocatechuate was significantly higher than that of vanillic acid. The above phenomena also exist in flavonoids. The inhibitory rate of flavonoids containing 7 and 4′ hydroxyl groups was significantly higher than if the 7 and 4′ hydroxyl groups are replaced. For example, the inhibitory activity of chrysoeriol was significantly higher than that of diosmetin, while the inhibitory activity of diosmetin was higher than that of 3′,5-dihydroxy-7-(*β*-D-glucopyranosyloxy)-4′-methoxyflavone and cofloxacin-7-O-*β*-D-glucoside. The inhibitory rate of flavonoids containing 3-hydroxy was significantly higher than that of compounds without 3-hydroxy or if the 3-hydroxy was replaced. For example, the inhibitory activity of quercetin was significantly higher than that of 4′-hydroxyscutellarin, but the inhibitory activity of 4′-hydroxyscutellarin was higher than that of quercetin-3-o-glucoside. Additionally, the inhibitory activity of koellin was higher than that of apigenin but lower than that of luteolin. It is suggested that the B-ring substituents, especially the hydroxyl group, can enhance the tyrosinase inhibitory activity, and the orthodihydroxy group of the B-ring had greater inhibitory activity [29].

## Figures and Tables

**Table 1:** Polar extracts of *Trichosanthes* pulp and the optimal concentration.

Groups	Polar extracts	C1 (mg/mL)	C2 (mg/mL)	C3 (mg/mL)	C4 (mg/mL)	C5 (mg/mL)
E1	100% Met	0.930	0.233	0.116	0.058	0.029
E2	75% (Met : EtoAC)	0.690	0.173	0.086	0.043	0.022
E3	50% (Met : EtoAC)	0.580	0.145	0.073	0.036	0.018
E4	25% (Met : EtoAC)	0.100	0.025	0.013	0.006	0.003
E5	10% (Met : EtoAC)	0.110	0.028	0.014	0.007	0.003
E6	5% (Met : EtoAC)	0.350	0.088	0.044	0.022	0.011
E7	100% EtoAC	0.380	0.095	0.048	0.024	0.012
E8	75% (EtoAC : Pet)	0.080	0.020	0.010	0.005	0.003
E9	50% (EtoAC : Pet)	0.060	0.015	0.008	0.004	0.002
E10	25% (EtoAC : Pet)	0.500	0.125	0.063	0.031	0.016
E11	10% (EtoAC : Pet)	1.170	0.293	0.146	0.073	0.037

**Table 2:** Summary of monomers.

Compound	Compound name	Reference
1	*p*-Hydroxybenzaldehyde	[13]
2	Salicylic acid	[14]
3	Vanillic acid	[15]
4	Isovanillic acid	[16]
5	Protocatechuate	[17]
6	*trans*-Cinnamic acid	[17]
7	4-Coumaric acid	[18]
8	*trans*-Ferulic acid	[15]
9	Drechslerol-B	[19]
10	Cyclotucanol 3-palmitate	[20]
11	5-Acetoxymethyl-2-furaldehyde	[21]
12	5-Hydroxymethylfurfural	[22]
13	Diosmetin	[23]
14	Apigenin	[23]
15	Chrysoeriol	[24]
16	Luteolin	[23]
17	4′-Hydroxyscutellarin	[22]
18	Quercetin	[25]
19	3′,5-Dihydroxy-7-(*β*-D-glucopyranosyloxy)-4′-methoxyflavone	[23]
20	Cofloxacin-7-O-*β*-D-glucoside	[24]

## Data Availability

The monomer data used to support the findings of this study are included within the article and supplementary information file.

## Supplementary Materials

Supplementary Materials The supplementary file is the 2H-NMR and 13C-NMR data of 20 compounds for readers to analyze and check the compounds.
