# Supplementary material for: Whitening Activity of Constituents Isolated from the Trichosanthes Pulp
Source: Evid Based Complement Alternat Med. 2020 Jul 22;2020:2582579. doi: 10.1155/2020/2582579 (PMC7396110; doi:10.1155/2020/2582579)
Supplement: Supplementary Materials — The supplementary file is the 2H-NMR and 13C-NMR data of 20 compounds for readers to analyze and check the compounds. [file 2582579.f1.docx]

Compound 1: white crystal, m.p.117-119℃; ESI-MS m/z 121[M-H]-, molecular weight is 122; ^1^H-NMR[400MHz, DMSO-*d*_6_] δ: 7.71(2H, d, H-2, 6), 6.89(2H, d, H-3, 5), 9.68(1H, s, -OH), 9.89(1H, s, -COH); ^13^C-NMR[100MHz, DMSO-*d*_6_] δ: 129.51(C-1), 132.72(C-2), 116.4(C-3), 164.32(C-4), 116.4(C-5), 132.72(C-6), 191.04(C-7). The above data are basically consistent with those reported in literature [13]. Therefore, the compound is identified as *p*-hydroxybenzaldehyde.

Compound 2: white crystal powder, m.p.158-161℃; ESI-MS m/z 137 [M-H]-; ^1^H-NMR[400MHz, DMSO-*d*_6_]δ: 7.08(1H, d, H-3), 7.58(1H, d, H-4), 7.51(1H, m, H-5), 8.03(1H, d, H-6), 12.04(1H, s, -COOH), 15.27(1H, s, -OH); ^13^C-NMR[100MHz, DMSO-*d*_6_] δ: 171.86(-COOH), 113.15(C-1), 162.23(C-2), 117.65(C-3), 135.37(C-4), 121.25(C-5), 131.78(C-6). The above data are basically consistent with those reported in literature [14]. Therefore, the compound is identified as salicylic acid.

Compound 3: white crystal, m.p.208-210℃; ESI-MS m/z, 167[M-H]-, molecular weight is168; ^1^H-NMR[400MHz, DMSO-*d*_6_] δ: 7.43(1H, s, H-2), 7.61(1H, d, H-6), 7.25(1H, d, H-5), 9.55(1H, s, -OH), 12.74(1H, s, -COOH), 3.83(3H, s, 3-OCH_3_); ^13^C-NMR[100MHz, DMSO-*d*_6_] δ: 169.34(-COOH), 123.85(C-1), 114.72(C-2), 149.15(C-3), 153.98(C-4), 114.16(C-5), 124.06(C-6), 56.15(-OCH_3_). The above data are basically consistent with those reported in literature [15]. Therefore, the compound is identified as vanillic acid.

Compound 4: white to light yellow crystalline powder, m.p.250-253℃; ESI-MS m/z 167 [M-H]-; ^1^H-NMR[400MHz, DMSO-*d*_6_] δ: 7.52(1H, s, H-2), 7.02(1H, d, H-5), 7.66(1H, d, H-6), 9.27(1H, s, -OH), 12.74(1H, s, -COOH), 3.86(3H, s, 3-OCH_3_); ^13^C-NMR[100MHz, DMSO-*d*_6_] δ: 169.34(-COOH), 123.91(C-1), 117.02(C-2), 147.15(C-3), 155.34(C-4), 112.16(C-5), 123.96(C-6), 56.15(-OCH_3_). The above data are basically consistent with those reported in literature [16]. Therefore, the compound is identified as isovanillic acid.

Compound 5: light yellow crystalline powder, m.p.198-200℃; ESI-MS m/z: 153[M-­H]-, molecular weight is 194; ^1^H-NMR[400MHz, DMSO-*d*_6_]δ: 7.35(1H, s, H-2), 7.06(1H, d, H-5), 7.49(1H, d, H-6), 9.48(2H, s, 34-OH), 12.74(1H, s, -COOH); ^13^C-NMR[100MHz, DMSO-*d*_6_] δ: 169.34(-COOH), 124.25(C-1), 117.42(C-2), 145.95(C-3), 152.58(C-4), 117.26(C-5), 124.36(C-6). The above data are basically consistent with those reported in literature [17]. Therefore, the compound is identified as protocatechuate.

Compound 6: white crystal, m.p.132-134℃; ESI-MS m/z 147 [M-H]-; ^1^H-NMR[400MHz, DMSO-*d*_6_] δ: 7.54(2H, d, H-2, 6), 7.38(2H, d, H-3, 5), 7.33(1H, s, H-4), 7.54(1H, d, H-7), 6.27(1H, d, H-8), 12.05(1H, s, -COOH); ^13^C-NMR[100MHz, DMSO-d_6_] δ: 135.22(C-1), 128.59(C-2, 6), 128.63(C-3, 5), 127.98(C-4), 144.24(C-7, C=C), 116.56(C-8, C=C), 171.55(C-9, -CO-). The above data are basically consistent with those reported in literature [17]. Therefore, the compound is identified as *trans*-cinnamic acid.

Compound 7: light yellow crystalline, m.p.212-214℃; ESI-MS m/z: 163 [M-H]-; ^1^H-NMR[400MHz, DMSO-*d*_6_] δ: 7.45(2H, d, H-2, 6), 6.59(2H, d, H-3, 5), 7.49(1H, d, H-7), 6.27(1H, d, H-8), 9.68(1H, s, -OH), 12.05(1H, s, -COOH); ^13^C-NMR[100MHz, DMSO-d_6_] δ: 127.85(C-1), 130.62(C-2, 6), 115.83(C-3, 5), 157.76(C-4), 144.24(C-7, C=C), 116.58(C-8, C=C), 171.55(C-9, -CO-). The above data are basically consistent with those reported in literature [18]. Therefore, the compound is identified as 4-coumaric acid.

Compound 8: light yellow crystalline, m.p.168-177℃; ESI-MS m/z 193 [M-H]-; ^1^H-NMR[400MHz, DMSO-*d*_6_]δ: 7.11(1H, s, H-2), 7.01(1H, d, H-5), 6.79(1H, d, H-6), 9.55(1H, s, -OH), 12.04(1H, s, -COOH), 7.45(1H, d, H-7, C=C), 6.27(1H, d, H-8, C=C), 3.83(3H, s, 3-OCH_3_); ^13^C-NMR[100MHz, DMSO-*d*_6_] δ: 171.56(-COOH), 127.65(C-1), 111.92(C-2), 149.15(C-3), 147.99(C-4), 116.86(C-5), 122.96(C-6), 56.15(-OCH_3_). The above data are basically consistent with those reported in literature [15]. Therefore, the compound is identified as *trans*-ferulic acid.

Compound 9: yellow oily liquid; ESI-MS m/z: 603 [M-H]-; ^1^H-NMR(400MHz, DMSO) δ: 4.49(1H, s, -OH), 2.38(2H, s, H-2), 2.35(2H, s, H-3), 5.43(2H, s, H-4、5), 2.16(2H, s, H-6), 1.26(s, H7-H19), 0.88(3H, s, H-20、20'), 4.06(2H, s, H-1') presumed to be -O-CH2-, 1.77(2H, s, H-2'), 3.40(1H, s, H-3'), 5.34(2H, s, H-11'-H12'), 2.16(4H, s, H-10'-H-13'), 1.26(s, H14'-H19'); ^13^C-NMR[100MHz, DMSO] δ: 173.42(C-1), 34.43(C-2), 28.71(C-3), 129.86(C-4), 131.3(C-5), 33.75(C-6), 29.9(C-7), 29.6(C8-C19), 14.18(C-20), 61.01(C-1'), 36.62(C-2'), 70.23(C-3'), 37.53(C-4'), 25.41(C-5'), 29.7(C6'-C9'), 27.72(C-10', 13'), 130.64(C11'-C12'), 29.35(C14'-C19'), 14.19(C-20'). The above data are basically consistent with those reported in literature [19]. Therefore, the compound is identified as drechslerol-B.

Compound 10: yellow oily liquid; ESI-MS m/z: 717.6 [M+Na]+; ^1^H-NMR(400MHz, DMSO) δ: 4.27(1H, t, H-3), 0.15-0.45(2H, t, H-19), 5.18-5.32(2H, t, 31-C=CH2), 4.77(1H, t, -OH), 2.35(2H, t, H-2'); ^13^C-NMR[100MHz, DMSO] δ: 31.63(C-1), 26.96(C-2), 80.45(C-3), 39.56(C-4), 47.22(C-5), 20.96(C-6), 25.83(C-7), 47.87(C-8), 20.29(C-9), 26.13(C-10), 26.55(C-11), 32.96(C-12), 45.44(C-13), 48.97(C-14), 35.60(C-15), 28.26(C-16), 52.41(C-17), 18.03(C-18), 29.86(C-19), 36.36(C-20), 18.44(C-21), 35.96(C-22), 28.04(C-23), 156.92(C-24), 73.68(C-25), 29.35(C-26), 29.35(C-27), 25.50(C-28), 15.24(C-29), 19.34(C-30), 106.77(C-31), 173.6(C-1'), 34.98(C-2'), 25.23(C-3'), 29.26(C-4'), 29.36(C-5'-C-13'), 31.96(C-14'), 22.76(C-15'), 14.15(C-16'). The above data are basically consistent with those reported in literature [20]. Therefore, the compound is identified as cyclotucanol 3-palmitate.

Compound 11: yellow flake crystals, m.p.54-56℃; ESI-MS m/z 167 [M-H]-; 1H-NMR[400MHz, DMSO-d6] δ: 7.42(1H, d, H-3), 6.73(1H, d, H-4), 5.09(2H, s, -CH2-O-), 2.09(1H, s, -CH3), 9.68(1H, s, -COH); 13C-NMR[100MHz, DMSO-d6] δ: 178.16(-COH), 153.12(C-2), 121.92(C-3), 111.65(C-4), 155.57(C-5), 170.21(-CO-), 60.15(-CH2-O-), 20.15(-CH3). The above data are basically consistent with those reported in literature [21]. Therefore, the compound is identified as 5-acetoxymethyl-2-furaldehyde.

Compound 12: beige crystalline powder, m.p.30-34℃; ESI-MS m/z 125 [M-H]-; ^1^H-NMR[400MHz, DMSO-*d*_6_] δ: 7.52(1H, d, H-3), 6.73(1H, d, H-4), 4.39(2H, s, -CH2-), 5.12(1H, s, -OH), 9.68(1H, s, -COH); ^13^C-NMR[100MHz, DMSO-*d*_6_] δ: 178.14(-COH), 153.12(C-2), 121.95(C-3), 111.64(C-4), 161.66(C-5), 57.05(O-CH2-). The above data are basically consistent with those reported in literature [22]. Therefore, the compound is identified as 5-hydroxymethylfurfural.

Compound 13: yellow crystalline, m.p.257-258℃; ESI-MS m/z 299 [M-H]-; ^1^H-NMR[400MHz, DMSO-d6] δ: 6.71(1H, d, H-3), 5.99(1H, s, H-6), 6.05(1H, s, H-8), 7.21(1H, d, H-2'), 6.76(1H, d, H-3'), 6.69(1H, d, H-6'), 12.97(1H, s, 5-OH), 10.18(1H, s, 7-OH), 9.17(1H, s, 5'-OH), 3.86(3H, s, 4'-CH3); ^13^C-NMR[100 MHz, DMSO-d6] δ: 163.65(C-2), 104.53(C-3), 182.12(C-4), 161.88(C-5), 98.37(C-6), 166.46(C-7), 94.03(C-8), 158.79(C-9), 104.42(C-10), 123.0(C-1'), 121.45(C-2'), 112.12(C-3'), 149.32(C-4'), 147.15(C-5'), 114.94(C-6'), 56.14(-O-CH3). The above data are basically consistent with those reported in literature [23]. Therefore, the compound is identified as diosmetin.

Compound 14: light yellow crystalline, m.p.346-348℃; ESI-MS m/z 269 [M-H]-; ^1^H-NMR[400MHz, DMSO-d6] δ: 6.71(1H, m, H-3), 5.99(1H, s, H-6), 6.05(1H, s, H-8), 7.48(1H, d, H-2'、6'), 6.65(1H, m, H-3'、5'), 12.97(1H, s, 5-OH), 10.18(1H, s, 7-OH), 9.68(1H, s, 5'-OH); ^13^C-NMR[100 MHz, DMSO-d6] δ: 163.65(C-2), 104.53(C-3), 182.12(C-4), 161.88(C-5), 98.37(C-6), 166.46(C-7), 94.03(C-8), 158.79(C-9), 104.42(C-10), 123.0(C-1'), 129.24(C-2'), 115.82(C-3'), 157.72(C-4'), 115.85(C-5'), 129.24(C-6'). The above data are basically consistent with those reported in literature [23]. Therefore, the compound is identified as apigenin.

Compound 15: yellow granular crystals, ESI-MS m/z 255 [M-H]-; ^1^H-NMR[400MHz, DMSO-d6] δ: 6.71(1H, s, H-3), 5.99(1H, s, H-6), 6.05(1H, s, H-8), 7.16(1H, d, H-2'), 6.99(1H, d, H-3'), 6.67(1H, s, H-6'), 12.97(1H, s, 5-OH), 10.18(1H, s, 7-OH), 9.55(1H, s, 4'-OH), 3.83(3H, s, -O-CH3); ^13^C-NMR[100 MHz, DMSO-d6] δ: 163.65(C-2), 104.53(C-3), 182.12(C-4), 161.88(C-5), 98.37(C-6), 166.46(C-7), 94.03(C-8), 158.79(C-9), 104.42(C-10), 122.72(C-1'), 121.54(C-2'), 116.82(C-3'), 147.92(C-4'), 149.14.85(C-5'), 111.94(C-6'), 56.15(-O-CH3). The above data are basically consistent with those reported in literature [24]. Therefore, the compound is identified as chrysoeriol.

Compound 16: yellow acicular crystals, m.p.328-330℃; ESI-MS m/z 285 [M-H]-; ^1^H-NMR[400MHz, DMSO-d6] δ: 6.71(1H, s, H-3), 5.99(1H, s, H-6), 6.05(1H, s, H-8), 6.89(1H, d, H-5'), 7.19(1H, m, H- 6'), 12.97(1H, s, 5-OH), 10.87(1H, s, 7-OH), 9.17(1H, s, 3'-OH), 9.45(1H, s, 4'-OH); ^13^C-NMR[100 MHz, DMSO-d6] δ: 163.65(C-2), 104.53(C-3), 182.12(C-4), 161.88(C-5), 98.37(C-6), 166.46(C-7), 94.03(C-8), 158.79(C-9), 104.42(C-10), 123.0(C-1'), 115.45(C-2'), 145.92(C-3'), 146.52(C-4'), 117.25(C-5'), 121.84(C-6'). The above data are basically consistent with those reported in literature [23]. Therefore, the compound is identified as luteolin.

Compound 17: yellow powder; ESI-MS m/z 286 [M-H]-; ^1^H-NMR[400MHz, DMSO-d6] δ: 6.71(1H, s, H-3), 5.85(1H, s, H-8), 7.48(2H, d, H-2'、6'), 6.65(2H, d, H-3'、5'), 12.97(1H, s, 5-OH), 12.97(1H, s, 5-OH), 8.73(1H, s, 6-OH), 9.48(1H, s, 6-OH), 9.68(1H, s, 4'-OH); ^13^C-NMR[100 MHz, DMSO-d6] δ: 163.62(C-2), 104.52(C-3), 182.12(C-4), 153.54(C-5), 143.92(C-6), 158.63(C-7), 95.43(C-8), 151.44(C-9), 106.92(C-10), 123.0(C-1'), 129.25(C-2'), 115.82(C-3'), 157.71(C-4'), 115.85(C-5'), 129.24(C-6'). The above data are basically consistent with those reported in literature [22]. Therefore, the compound is identified as 4'-hydroxyscutellarin.

Compound 18: yellow-green acicular crystals, m.p.313-314℃; ESI-MS m/z 301 [M-H]-; ^1^H-NMR[400MHz, DMSO-d6] δ: 5.94(1H, s, H-6), 6.05(1H, s, H-8), 7.04(1H, d, H-2'), 6.82(1H, d, H-3'), 6.52(1H, s, H- 6'), 10.68(1H, s, 3-OH), 12.97(1H, s, 5-OH), 10.17(1H, s, 7-OH), 9.48(1H, s, 4'-OH), 9.48(1H, s, 5'-OH); ^13^C-NMR[100 MHz, DMSO-d6]δ: 146.93(C-2), 136.52(C-3), 176.12(C-4), 161.88(C-5), 98.37(C-6), 166.46(C-7), 94.03(C-8), 158.79(C-9), 104.42(C-10), 123.0(C-1'), 121.85(C-2'), 117.25(C-3'), 146.52(C-4'), 145.95(C-5'), 115.34(C-6'). The above data are basically consistent with those reported in literature [25]. Therefore, the compound is identified as quercetin.

Compound 19: yellow crystalline, 253-255℃; ESI-MS m/z 461 [M-H]-; ^1^H-NMR[400MHz, DMSO-d6]δ: 6.71(1H, m, H-3), 6.17(1H, s, H-6), 6.74(1H, s, H-8), 7.21(1H, d, H-2'), 6.76(1H, m, H-3'), 6.69(1H, m, H-6'), 12.97(1H, s, 5-OH), 9.27(1H, s, 5'-OH), 3.86(3H, t, 4'-CH3), 5.80(1H, d, H-1'') are the signals from the glucose terminal carbon atom, 4.77(1H, s, 2''-OH), 4.71(1H, s, 3''-OH), 4.88(1H, s, 4''-OH), 3.94(H, s, 6''-OH) is a signal from glucose hydroxyl, ^13^C-NMR[100 MHz, DMSO-d6] δ: 163.65(C-2), 104.53(C-3), 182.12(C-4), 161.88(C-5), 98.12(C-6), 164.03(C-7), 92.93(C-8), 158.07(C-9), 103.42(C-10), 122.78(C-1'), 121.45(C-2'), 112.12(C-3'), 149.32(C-4'), 147.15(C-5'), 114.94(C-6'), 56.14(-O-CH3), 109.23(C-1'') are the signals from the glucose terminal carbon atom, 73.49(C-2''), 76.88(C-3''), 71.56(C-4''), 81.53 (C-5''), 62.21(C-6''). The above data are basically consistent with those reported in literature [23]. Therefore, the compound is identified as 3',5-dihydroxy-7-(β-D-glucopyranosyloxy)-4'-methoxyflavone.

Compound 20: yellow powder, 177-179℃; ESI-MS m/z: 463[M+H]+, 461[M-­H]-, molecular weight is 462; 1H-NMR[400MH, DMSO-d6] δ: 6.71(1H, m, H-3), 6.17(1H, s, H-6), 6.74(1H, s, H-8), 7.16(1H, d, H-2'), 6.99(1H, m, H-3'), 6.67(1H, m, H-6'), 12.97(1H, s, 5-OH), 9.55(1H, s, 4'-OH), 3.83(3H, t, -O-CH3), 5.80(1H, t, H-1'') are the signals of hydrogen on the carbon atom of glucose end group, 4.77(1H, s, 2''-OH), 4.71(1H, s, 3''-OH), 4.88(1H, s, 4''-OH); 3.94(1H, t, H-6''); 13C-NMR[100MHz, DMSO-d6]δ: 163.65(C-2), 104.53(C-3), 182.12(C-4), 161.88(C-5), 98.13(C-6), 164.02(C-7), 92.93(C-8), 158.03(C-9), 103.42(C-10), 122.62(C-1'), 121.54(C-2'), 116.82(C-3'), 147.92(C-4'), 149.14.85(C-5'), 111.94(C-6'), 56.15(-O-CH3), 109.23(C-1'') are the signals from the glucose terminal carbon atom, 73.43(C-2''), 76.88(C-3''), 71.52(C-4''), 81.53(C-5''), 62.21(C-6''). The above data are basically consistent with those reported in literature [24]. Therefore, the compound is identified as cofloxacin-7-O-β-D-glucoside.
